# Immunogenicity and Cross-Protective Efficacy Induced by an Inactivated Recombinant Avian Influenza A/H5N1 (Clade 2.3.4.4b) Vaccine against Co-Circulating Influenza A/H5Nx Viruses

**DOI:** 10.3390/vaccines11091397

**Published:** 2023-08-22

**Authors:** Sara H. Mahmoud, Ahmed A. Khalil, Noura M. Abo Shama, Marwa F. El Sayed, Reem A. Soliman, Naglaa M. Hagag, Nahed Yehia, Mahmoud M. Naguib, Abdel-Sattar Arafa, Mohamed A. Ali, Mounir M. El-Safty, Ahmed Mostafa

**Affiliations:** 1 Center of Scientific Excellence for Influenza Viruses, National Research Centre, Giza 12622, Egypt; su.hussein@nrc.sci.eg (S.H.M.); ma.alii@nrc.sci.eg (M.A.A.); 2 Veterinary Serum and Vaccine Research Institute, Agricultural Research Center (ARC), Abbasia, Cairo 11381, Egypt; ahme_2001@hotmail.com; 3 Central Laboratory for Evaluation of Veterinary Biologics, Agricultural Research Center (ARC), Abbasia, Cairo 11517, Egyptmelsafty_hobs@yahoo.com (M.M.E.-S.); 4 Reference Laboratory for Veterinary Quality Control on Poultry Production, Animal Health Research Institute, Agriculture Research Center, Giza 12618, Egypt; 5 Zoonosis Science Center, Department of Medical Biochemistry and Microbiology, Uppsala University, 75121 Uppsala, Sweden; 6 Texas Biomedical Research Institute, San Antonio, TX 78227, USA

**Keywords:** highly pathogenic avian influenza, immunogenicity, vaccine, AIV H5N1, AIV H5N8, clade 2.3.4.4b

## Abstract

Controlling avian influenza viruses (AIVs) is mainly based on culling of the infected bird flocks or via the implementation of inactivated vaccines in countries where AIVs are considered to be endemic. Over the last decade, several avian influenza virus subtypes, including highly pathogenic avian influenza (HPAI) H5N1 clade 2.2.1.2, H5N8 clade 2.3.4.4b and the recent H5N1 clade 2.3.4.4b, have been reported among poultry populations in Egypt. This demanded the utilization of a nationwide routine vaccination program in the poultry sector. Antigenic differences between available avian influenza vaccines and the currently circulating H5Nx strains were reported, calling for an updated vaccine for homogenous strains. In this study, three H5Nx vaccines were generated by utilizing the reverse genetic system: rgH5N1_2.3.4.4, rgH5N8_2.3.4.4 and rgH5N1_2.2.1.2. Further, the immunogenicity and the cross-reactivity of the generated inactivated vaccines were assessed in the chicken model against a panel of homologous and heterologous H5Nx HPAIVs. Interestingly, the rgH5N1_2.3.4.4 induced high immunogenicity in specific-pathogen-free (SPF) chicken and could efficiently protect immunized chickens against challenge infection with HPAIV H5N1_2.3.4.4, H5N8_2.3.4.4 and H5N1_2.2.1.2. In parallel, the rgH5N1_2.2.1.2 could partially protect SPF chickens against infection with HPAIV H5N1_2.3.4.4 and H5N8_2.3.4.4. Conversely, the raised antibodies to rgH5N1_2.3.4.4 could provide full protection against HPAIV H5N1_2.3.4.4 and HPAIV H5N8_2.3.4.4, and partial protection (60%) against HPAIV H5N1_2.2.1.2. Compared to rgH5N8_2.3.4.4 and rgH5N1_2.2.1.2 vaccines, chickens vaccinated with rgH5N1_2.3.4.4 showed lower viral shedding following challenge infection with the predefined HPAIVs. These data emphasize the superior immunogenicity and cross-protective efficacy of the rgH5N1_2.3.4.4 in comparison to rgH5N8_2.3.4.4 and rgH5N1_2.2.1.2.

## 1. Introduction

Since late 2020, outbreaks of highly pathogenic avian influenza (HPAI) H5N1 virus (clade 2.3.4.4b) continue to be reported around the world in both wild birds and, on several occasions, domestic poultry, with substantial genetic evolution and reassortment, enabling the emergence of several variants [1,2,3,4]. In late 2021, the HPAI H5N1 virus was found in migratory birds in Egypt with some cases in domestic poultry in early 2022 [5].

Avian influenza (AIV) infection control occurs predominantly via culling of infected poultry or through vaccination using conventional inactivated vaccines that genetically and antigenically match the circulating virus [6,7]. In 2017, the vaccine regime against H5 was updated in Egypt in response to the introduction of the HPAI H5N8 virus (clade 2.3.4.4b) [8]. With the introduction of the HPAI H5N1 virus (clade 2.3.4.4b) into the Egyptian poultry sector, the situation became more complicated due to the reported outbreaks of the HPAI H5N8 virus [9]. In addition, the HPAI H5N1 (clade 2.2.1) was primarily reported in Egypt in 2006 and declared to be an endemic viral pathogen in poultry in 2008. Since 2006, the HPAIV H5N1 (clade 2.2.1) has been subjected to continuous evolution events, ending up with the HPAI H5N1 virus of clade 2.2.1.2 that led to massive losses in poultry and zoonotic infections among human populations [10,11]. Despite this, H5N1 (clade 2.2.1.2) has not been reported since 2017, probably due to the application of a locally engineered inactivated H5N1 (clade 2.2.1.2) vaccine that matches the circulating strains and its replacement with HPAIV H5N8 (2.3.4.4b), it is worth mentioning that this subtype may reemerge, causing the situation to worsen.

Currently, the most commonly used avian influenza vaccines for poultry in Egypt are based on reverse genetics technology (rg) of influenza viruses by generating a reassortant six plus two candidate vaccine strain that comprises the surface glycoproteins, hemagglutinin (HA) and neuraminidase (NA), of the circulating HPAI H5 virus and the other six segments from a cell-culture- and embryonated-egg-adapted influenza A/Puerto Rico/8/34 (H1N1; PR8) virus [12,13,14].

The cocirculation of the Eurasian-origin HPAI H5N8 virus (clade 2.3.4.4b) and other H5Nx viruses is having a negative impact on the poultry industry as well as posing a threat to public health [15]. Until now, no or only few studies have investigated the immunogenicity and cross-protective efficacy of inactivated PR8-based avian influenza H5N1 (clade 2.3.4.4b) vaccine against other cocirculating H5Nx Viruses (clades 2.2.1.2 and 2.3.4.4b) in chickens and vice versa. In this study, recombinant PR8-based vaccines for H5Nx viruses (H5N1 clade 2.2.1.2 and H5N8 2.3.4.4b) were generated and evaluated for their immunogenicity and cross-protectiveness following challenge infections with homogeneous or heterologous HPAI H5Nx subtype(s) of various clades.

## 2. Materials and Methods

### 2.1. Cells, Specific Pathogen-Free (SPF) Embryonated Eggs and Chicks, and Viruses

Specific pathogen-free (SPF) embryonated chicken eggs (day 1) and SPF chicks (two weeks old) were obtained from Koum Oshiem SPF Chicken Farm, Fayoum, Egypt. The MDCK and 293T cells were kindly provided from the cell culture collection of the National Research Centre (NRC), Egypt. The HPAI viruses (HPAIVs) of clade 2.3.4.4, A/chicken/Egypt/F71-F114C/2022(H5N1) (HPAIV_H5N1_2.3.4.4) and A/chicken/Egypt/F71-S86C/2022(H5N8) (HPAIV_H5N8_2.3.4.4) were collected within a current active surveillance study to monitor the currently circulating avian influenza viruses in Egypt. Both viruses were propagated in SPF embryonated chicken eggs and titrated using plaque infectivity assay and TCID_50_ as previously described [16,17].

### 2.2. Phylogenetic Analysis

To address the phylogenetic relatedness between the Egyptian H5Nx involved in this study, representative HPAI H5N8 strains from clade 2.3.4.4a and 2.3.4.4b, H5N1 strains from clade 2.3.4.4b and HPAI H5N1 strains of clade 2.2.1 were retrieved from the GISAID platform (GISAID, http://www.gisaid.org, last accessed 3 January 2023). Representative viruses were selected based on geographical locations for both HA and NA gene segments. Retrieved sequences were then aligned using MAFFT [18] implemented in Geneious Prime 2022.2.2 (https://www.geneious.com). Phylogenetic trees were performed using maximum likelihood methodology based on Akaike criterion in IQ-tree software version 1.1.3 [19] after the selection of the best-fitted model. Phylogenetic trees were annotated and viewed using FigTree v1.4.2 software (http://tree.bio.ed.ac.uk/software/figtree/, accessed on 5 January 2023) and Inkscape V1.1 (https://inkscape.org, accessed on 5 January 2023).

### 2.3. Construction of Reverse Genetics Plasmids

The highly pathogenic avian influenza viruses of clade 2.3.4.4, A/chicken/Egypt/F71-F114C/2022 (H5N1) (HPAIV_H5N1_2.3.4.4) and A/chicken/Egypt/F71-S86C/2022(H5N8) (HPAIV_H5N8_2.3.4.4) were used to amplify the monobasic HA and NA segments of each strain using gene-specific primers and cloned into pMKPccdB plasmid as previously described [20] to construct pMP-HA_H5N8_2.3.4.4, pMP-NA_H5N8_2.3.4.4, pMP-HA_H5N1_2.3.4.4 and pMP-NA_H5N1_2.3.4.4. The HA and NA segments of H5N1_2.2.1.2 were previously cloned into pMPccdB vector “pMP-HA_H5N1_2.2.1.2 and pMP-HA_H5N1_2.2.1.2” [21] and were directly used to convert the multibasic cleavage site-encoding sequence into monobasic cleavage site-encoding sequence. The multibasic amino acids at the hemagglutinin cleavage sites of H5N1_2.3.4.4, H5N8_2.3.4.4_,_ and H5N1_2.2.1.2 viruses were engineered into a monobasic form (PQIETR/GLF) as described previously [12].

### 2.4. Rescue and Propagation of PR8-Based Candidate Vaccines against HPAI H5N1_2.3.4.4, HPAI H5N8_2.3.4.4, and HPAI H5N1_2.2.1.2 Viruses

Using reverse genetic technology of IAVs, the constructed HA- and NA-encoding plasmids of each strain were transfected into 293T/MDCK coculture together with the bidirectional plasmids flanking the 6 internal protein-encoding segments from the egg- and cell-culture-adapted influenza A/PR/8/34(H1N1) virus to generate high yield PR8-based experimental vaccines [22]. Rescued reassortant PR8-based low pathogenic candidate vaccine strains, rgH5N1_2.3.4.4, rgH5N8_2.3.4.4 and rgH5N1_2.2.1.2, were individually inoculated into 11-day-old specific pathogen-free embryonated chicken eggs (SPF-ECE). The allantoic fluids were harvested at 24 and 36 h post infection and titrated using EID_50_ and HA assays.

HA titers of the rgH5N1_2.3.4.4, rgH5N8_2.3.4.4 and rgH5N1_2.2.1.2 were adjusted individually using phosphate buffered saline to 7 log_2_ HA/25 μL. The rgH5N1_2.3.4.4, rgH5N8_2.3.4.4 and rgH5N1_2.2.1.2 were further inactivated by adding 0.1% formalin overnight. To verify the inactivation process, an aliquot of 100 µL of each inactivated virus was inoculated separately into MDCK cells and 11-day-old SPF-ECE for three successive passages. The inactivated candidate vaccine strains “antigens” were mixed with adjuvant Montanide ISA 71 VG (Seppic Inc., Puteaux, France) in the ratio of 30% antigen to 70% adjuvant as recommended by the manufacturer, followed by homogenization for 3 min on ice using a mixer homogenizer to form a stable water in oil (W/O) emulsion as a vaccine formulation. To test the safety of the formulated, 5 SASSO chickens (3-week-old) were individually inoculated with a double dose volume (1 mL) of each candidate vaccine via intramuscular route. The inoculated SASSO chickens were monitored for any symptoms or local lesions at the vaccine administration site for 2 weeks post inoculation.

### 2.5. Immunization of SPF Chickens and Seroconversion Determination

To investigate the effectiveness of the propagated vaccines, one hundred and sixty 1-day-old SPF chickens were randomly divided into four groups (G1–G4), 40 for each. Two weeks later SPF chicken in G1–G3 were immunized with 10^8.5 EID_50_ rgH5N1_2.3.4.4, rgH5N8_2.3.4.4 and rgH5N1_2.2.1.2 vaccines in a total volume of 0.3 mL via intramuscular (IM) route, respectively. Whereas G4, representing the unvaccinated control group, received 0.3 mL of phosphate-buffered saline (PBS; pH 7.4) via IM route and was kept as a negative control. Blood samples were obtained at 14-, 21- and 28-days post immunization (dpi) from all groups. In addition, sera were retrieved and subjected to hemagglutination inhibition assay (HAI) (WHO, 2002) to assess the seroconversion and respective antibody titers. To explore the cross-reactivity of the provoked antibodies against homologous and heterologous HPAIVs of H5-subtype, collected sera samples were tested via HAI against HPAIV H5N1_2.3.4.4, H5N8_2.3.4.4 and H5N1_2.2.1.2.

### 2.6. Challenge Infection and Viruses Shedding

Virus challenge was conducted on 4th week post-vaccination (wpv) at the Central Laboratory for Evaluation of Veterinary Biologics, Agricultural Research Center, (ARC), Abbasia, Cairo, Egypt. A total of 10 SPF chickens from each of G1, G2 and G3, were vaccinated with rgH5N1_2.3.4.4, rgH5N8_2.3.4.4 and rgH5N1_2.2.1.2, respectively, were infected with HPAIV_H5N1_2.3.4.4, HPAIV_H5N8_2.3.4.4 and HPAIV_H5N1_2.2.1.2 viruses. Chickens from G4 were subjected to challenge infections with the three HPAIVs (5 chickens for each virus). The challenge dose for each virus was 10^6^ EID_50_/0.1 mL and was inoculated via intranasal route. Following challenge infection, birds were daily monitored for clinical signs and mortality for 10 days post infection (dpi). The protectiveness of the vaccine was calculated according to the following equation:

$$\mathrm{Protection}\;\left(\%\right)=\frac{\mathrm{Number}\;\mathrm{of}\;\mathrm{survivals}}{\mathrm{Total}\;\mathrm{number}\;\mathrm{of}\;\mathrm{challenged}\;\mathrm{birds}}\times 100$$

To determine viral shedding, oropharyngeal (OP) and cloacal swabs were obtained from each bird on 3rd, 5th and 7th day post infection, then titrated by real-time RT-PCR using gene-specific primers as previously described [17].

### 2.7. Biosafety and Ethical Approval

All experiments including infectious viruses were performed in negative pressure-based biosafety level 3 cabinets and isolators at the Central Laboratory for Evaluation of Veterinary Biologics (CLEVB), Egypt (PLAS LABS, Lansing, MI, USA) and the National Research Centre (NRC), Egypt. All animal experiments were conducted in accordance with the Declaration of Helsinki and approved by the Medical Research Ethics Committee (MREC) of the NRC, Egypt (approval code: 19–274). All experiments involving IAVs were performed using Biosafety Level 2 and 3 laboratories and isolators approved for such use by the local authorities at CLEVB and NRC, Egypt.

### 2.8. Statistical Analyses

All statistical analyses were carried out using GraphPad Prism V5 software (GraphPad Inc., San Diego, CA, USA). Repeated measures ANOVA followed by Bonferroni post-hoc test were used to compare virus titers. The significant differences are indicated (** = *p* < 0.01, *** = *p* < 0.001 and non-significant = ns).

## 3. Results

### 3.1. Phylogenetic Analysis of the Candidate Vaccine Strains

**Figure 1:**
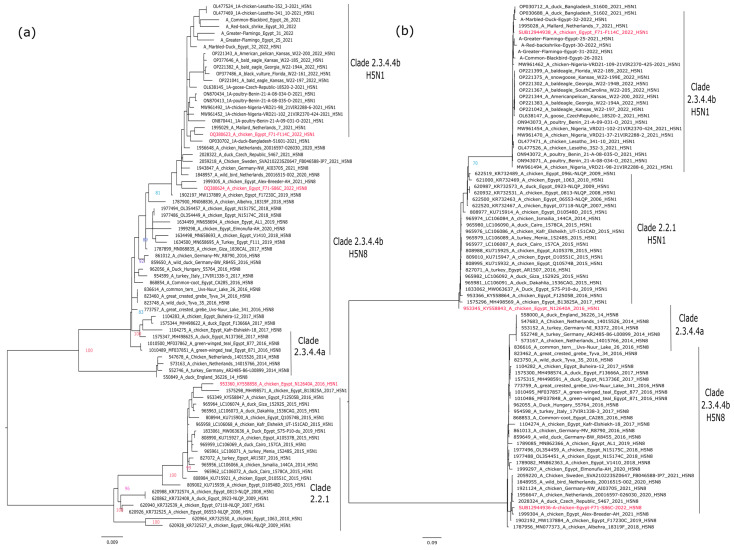
Phylogenetic analysis of the surface glycoproteins of the selected candidate vaccine strains versus different circulating isolates of their corresponding clades. (**a**) Phylogenetic tree of the HA of gene segment of representative strains of H5 gene; (**b**) Phylogenetic tree of the NA gene segment of representative strains (N1 and N8) gene of the selected candidate vaccine strains. Phylogenetic trees were performed using maximum likelihood methodology based on Akaike criterion with 1000 ultrafast bootstrap replicates in IQ-tree software version 1.1.3 [19] after the selection of the best-fitted model (GTR + F + G4 for both HA and NA). Selected candidate vaccine strains are shown in red.

The topology of the phylogenetic trees of the HA gene segments revealed that the Egyptian H5Nx strains are located within three different groups. The HA of influenza A/chicken/Egypt/F71-S86C/2022(H5N8) and A/chicken/Egypt/F71-F114C/2022(H5N1) indicated that both viruses are of Eurasian origin and belong to highly pathogenic avian influenza A(H5N1), belonging to the Gs/GD lineage, clade 2.3.4.4b. Both viruses are closely related to influenza A/H5N1 strains recently detected in Africa and Europe. On the other hand, influenza A/chicken/Egypt/N12640A/2016(H5N1) is located within clade 2.2.1, which is similar to the H5N1 viruses circulating in Egypt from 2006 to 2017 (Figure 1a).

The same phylogenetic clustering was found for the NA N1 gene segment of influenza A/chicken/Egypt/F71-F114C/2022(H5N1) and A/chicken/Egypt/N12640A/2016 (H5N1), which is highly distinct from the NA N8 of influenza A/chicken/Egypt/F71-S86C/2022(H5N8) (Figure 1b).

### 3.2. Generation of the Candidate H5-Type Vaccine Strains

**Figure 2:**
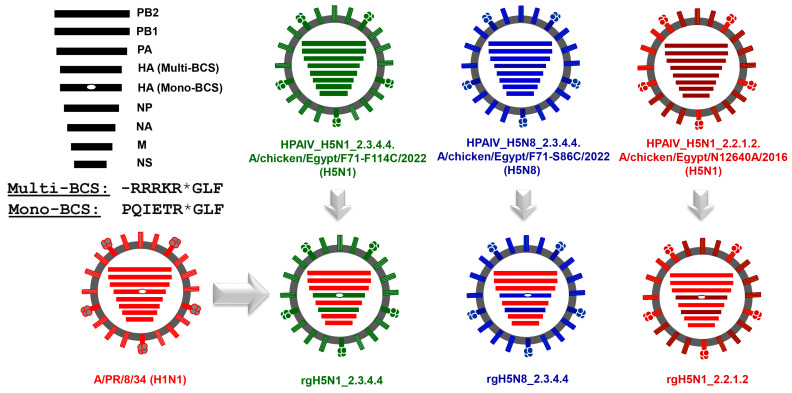
Genetic constellation of the generated candidate vaccine strains against different H5Nx strains. The HA_Multi-BCS: Multibasic Cleavage Site (highly pathogenic) and HA_Mono-BCS: Monobasic Cleavage Site (low pathogenicity). The asterisk symbol (*) denotes the cleavage position.

Following the cloning of the HA and NA from influenza A/chicken/Egypt/F71-F114C/2022(H5N1), A/chicken/Egypt/N12640A/2016(H5N1) and A/chicken/Egypt/F71-S86C/2022(H5N8) into specific reverse genetics vectors, namely pMKP*ccd*B [12], the candidate PR8-based vaccine strains were rescued by generating PR8 reassortants expressing the surface glycoproteins (HA and NA) of the corresponding predefined avian influenza viruses and the other internal protein-encoding segments from the cell-culture and egg-adapted PR8 strain (Figure 2).

**Figure 3:**
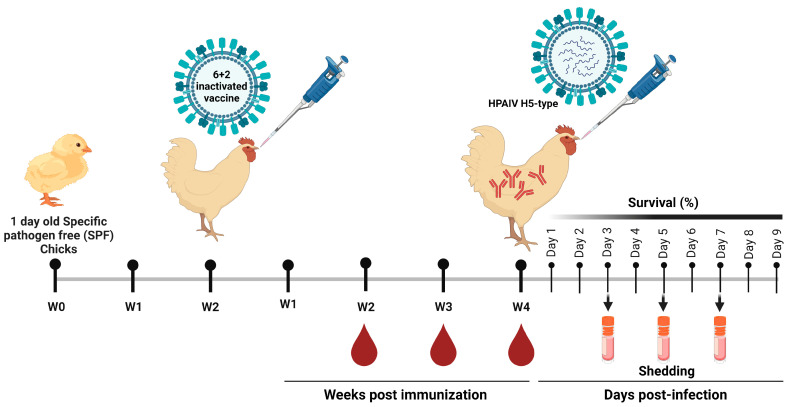
Study design including vaccination and sampling time points. The SPF chicks were immunized with the formulated vaccines against the control PBS group at 2-weeks old and samples were collected at the second-week post immunization (WPI). The immunized and control chicks were subjected to challenge infections with homologous and heterologous HPAI H5N1 and H5N8 viruses at 4th WPI. The challenged chicks were monitored for 9 days post challenge for mortality and viral shedding.

To mitigate the pathogenicity of the PR8-based vaccine strains and support their productive replication in embryonated eggs, the multibasic cleavage sites in the three strains were converted in the cloned HA segments using specific mutagenesis primers into monobasic cleavage sites (Figure 3).

Following virus rescue, the PR8-based vaccine strains, rgH5N1_2.3.4.4, rgH5N8_2.3.4.4 and rgH5N1_2.2.1.2, were propagated in embryonated eggs and the allantoic fluids were titrated using EID_50_ and HA assay (data not shown).

The titrated allantoic fluids of the different PR8-based vaccine strains were completely inactivated using 0.1% formalin and the inactivation process was controlled by negative cytopathic effect or HA titers for the supernatants or allantoic fluids of MDCK cell cultures or embryonated eggs that were inoculated with the inactivated candidate vaccine strains. The amount of residual-free formaldehyde in formalin-inactivated vaccine was neutralized with sodium bisulfite (0.2%) for 10 min. Subsequently, the inactivated candidate vaccines were adjuvanted with Montanide ISA 71 in the ratio of 30% antigen and 70% adjuvant and formulated via homogenization into W/O emulsion.

The one-day specific pathogen free (SPF) chicks were then caged for two weeks to deplete residual maternal antibodies. The SPF chicks were then immunized with 300 µL (10^8.5 EID_50_) of the formulated rgH5N1_2.3.4.4, rgH5N8_2.3.4.4 and rgH5N1_2.2.1.2 vaccines.

### 3.3. Immunogenicity of the Formulated H5-Type Vaccine and Cross-Reactivity against Homologous and Heterologous HPAI H5-Type Strains

**Figure 4:**
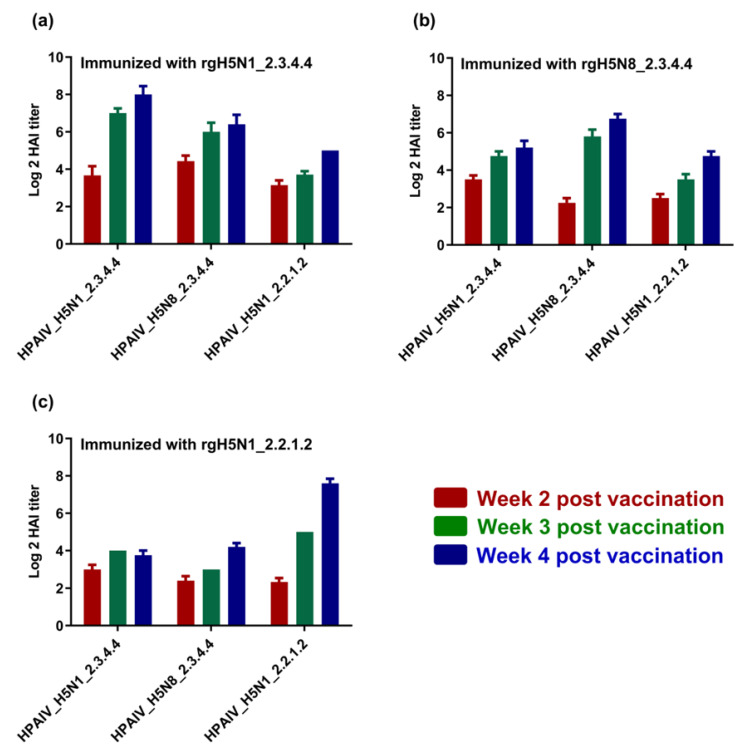
Immunogenicity of the formulated H5-type vaccine and cross-reactivity against homologous and heterologous HPAI H5-type strains. Sera from immunized chicks using (**a**) rgH5N1_2.3.4.4; (**b**) rgH5N8_2.3.4.4; and (**c**) rgH5N1_2.2.1.2, were tested using HAI against different H5-type strains of clades 2.3.4.4 and 2.2.1.2.

To investigate the immunogenicity and the cross-reactivity of antibodies raised against each candidate vaccine of the three inactivated vaccine strains, sera samples that were collected from chicks at the second, third and fourth WPI were titrated for the elicited antibodies via hemagglutination inhibition assay (HAI) using either homologous or heterologous HPAI H5-type strains. Interestingly, different vaccines showed high immunogenicity and cross-reactivity with heterologous HPAI H5-type strains (Figure 4a–c). Nevertheless, the raised antibodies for each vaccine were also shown to interact with and neutralize homologous HPAI H5-type strains to comparable or higher levels when compared to those against heterologous HPAI H5-type strains (Figure 4a–c).

### 3.4. Protectiveness of the Formulated Vaccines against HPAI H5-Type Viruses and Survival of Immunized Chickens following Homologous and Heterologous Challenge Infection

**Figure 5:**
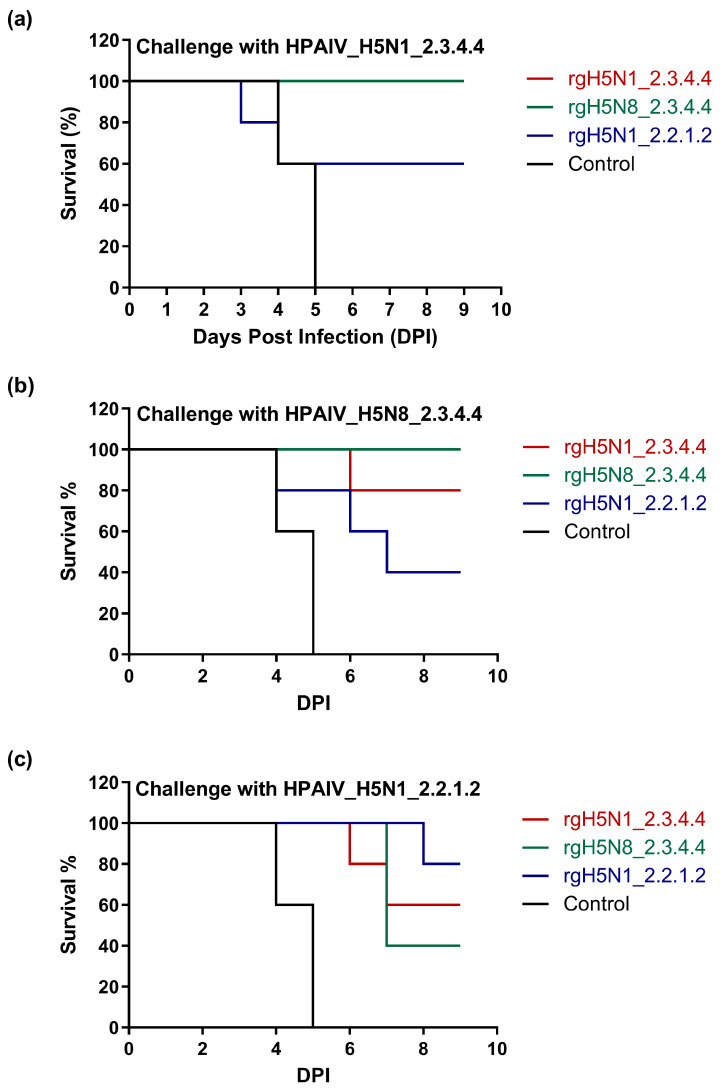
Survival rate of vaccinated and unvaccinated chickens following challenge with homologous and heterologous HPAI H5-type strains. Immunized chickens were challenged intranasally (10^6^ EID_50_/0.1 mL) with (**a**) HPAIV_H5N1_2.3.4.4, (**b**) HPAIV_H5N8_2.3.4.4 and (**c**) HPAIV_H5N1_2.2.1.2.

Challenge infection of vaccinated and control non-vaccinated chickens was achieved with the three HPAI H5Nx viruses of clade 2.3.4.4b and 2.2.1.2 (Table 1). Vaccine protection based on the percent bird survival varied greatly among the applied homologous and heterologous challenge viruses. We observed a survival efficacy rate of 80–100% for rgH5N1_2.3.4.4 and rgH5N8_2.3.4.4 vaccine strains versus HPAI challenge viruses of the same clade, namely HPAIV_H5N1_2.3.4.4 and HPAIV_H5N8_2.3.4.4 (Table 1 and Figure 5a,b), but these protection percentages were reduced to 40–60% when challenged with HPAIV_H5N1_2.2.1.2 (Table 1 and Figure 5c). In contrast, a survival rate of 80% was observed for rgH5N1_2.2.1.2-immunized chicken when challenged with homologous HPAI strain and 40% or 60% after challenge infection with HPAIV_H5N8_2.3.4.4 and HPAIV_H5N1_2.3.4.4, respectively (Table 1 and Figure 5a–c).

### 3.5. Immunized Chickens with rgH5N1_2.3.4.4 Showed Limited Shedding following Challenge Infection with Homologous and Heterologous HPAI H5Nx Strains

**Figure 6:**
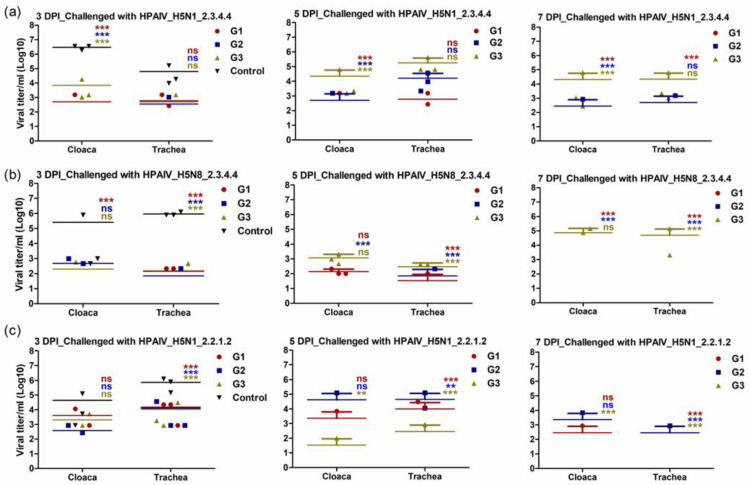
Viral shedding of the immunized chickens at 3-, 5- and 7-days post challenge infections (DPI). The immunized chicken with rgH5N1_2.3.4.4 (G1), rgH5N8_2.3.4.4 (G2) and rgH5N1_2.2.1.2 (G3) were subjected to challenge infections with homologous and heterologous HPAIV H5-type viruses; (**a**) HPAI_H5N1_2.3.4.4; (**b**) HPAI_H5N8_2.3.4.4 and (**c**) HPAI_H5N1_2.2.1.2. The significant differences, compared to the infected unvaccinated control “Control” are indicated (** = *p* < 0.01, *** = *p* < 0.001 and non-significant = ns) and highlighted in similar color to each corresponding group.

To investigate the capability of the different vaccines to control viral shedding after active challenge infection with homologous and heterologous HPAI H5Nx strains, virus shedding was detected at days three, five and seven post-infection (dpi). The OP and cloacal swabs from all the birds in the vaccinated and control groups were assessed for viral shedding. Independent of the challenging virus, all immunized chickens showed shedding titers that are lower than the control group at third dpi in both OP and cloacal samples (Figure 6a–c). Due to the fact that the control unvaccinated group showed up to 100% mortality at the fifth dpi, we compared the viral shedding at days five and seven post challenge for the vaccinated groups with the control group at the third dpi in both OP and cloacal samples. Interestingly, chickens that were vaccinated and challenged with a matching virus and clade had either no or very low levels of virus shedding at days five and seven, respectively (Figure 6a–c). Moreover, the findings of this experiment showed that vaccinated chickens with rgH5N1_2.3.4.4 displayed significantly lower OP titers than rgH5N8_2.3.4.4- and rgH5N1_2.2.1.2-vaccinated chickens following challenge infection with heterologous HPAI H5 virus-type strains (Figure 6a–c).

## 4. Discussion

The HPAI H5N1 virus, namely influenza A/Goose/Guangdong/1/96 (Gs/GD) lineage, has emerged firstly in 1996 in the poultry sector of China and was subsequently transmitted worldwide via migratory birds, causing poultry outbreaks and human infections [23,24,25]. With the extensive circulation of these H5-type viruses and the accumulation of a substantial number of non-silent adaptive mutations in the surface proteins “antigenic drift”, the Gs/Gd lineage viruses were differentiated into nine main clades with several subclades [26]. The H5Nx viruses of clade 2.3.4.4 with different emerging subclades (a–h) are frequently being reported [27,28]. Out of these different subclades, the H5N8 of 2.3.4.4b subclade has been globally established in the poultry sector leading to remarkably devastating outbreaks in poultry in Europe, Africa and Asia since its vast emergence in 2014 [9,29,30]. Due to a genetic reassortment of H5N8 (clade 2.3.4.4b) with other avian influenza viruses, a new H5N1 variant of clade 2.3.4.4b has emerged in Europe in late 2020 to subsequently become the most predominant AI strain causing extensive outbreaks in poultry and wild birds across Europe by the end of 2021 [31,32].

The H5N1 Gs/Gd lineage viruses (clade 2.2) emerged in Egypt in late 2005 and were declared to be endemic in 2008 due to the elevated numbers in poultry outbreaks and human zoonotic events [10]. Since then, the virus has been subjected to different evolution-driving pressures such as improperly imported vaccines and consequently differentiated into many clades including 2.2.1, 2.2.1.1, 2.2.1.1a and 2.2.1.2 [10]. Being a bridge between the three old world continents, Europe, Asia and Africa, where internationally important migration routes for birds intersect, enabling the migrating birds to disseminate new viruses to the Egyptian environment; by 2017, the predefined H5N1 variants were gradually replaced with the HPAIV H5N8 of clade 2.3.4.4 that emerged in Egypt in 2016 [33]. In late 2021, the newly emerging H5N1 of clade 2.3.4.4 has been introduced to Egypt via migratory birds and is currently cocirculating with the HPAIV H5N8 of clade 2.3.4.4 [5]. Due to the endemic situation of avian influenza viruses, the control strategy of avian influenza in Egypt recommends the application of inactivated vaccines that match genetically and antigenically to the circulating strains [34,35]. Being of the same clade, no studies have investigated the immunogenicity of inactivated H5N1_2.3.4.4 vaccine in poultry and/or cross-reactivity against other cocirculating H5-type strains and vice versa.

Compared to other therapeutic interventions, vaccination remains the most effective strategy to prevent viral disease and minimize public health hazards. Antigenic matching between the candidate vaccine strain and the circulating or contemporary viruses is fundamental for proper vaccine efficiency [36]. The current epidemiological situation worldwide and specifically in Egypt has led us to generate three recombinant H5-type vaccine forms including rgH5N1_2.3.4.4, rgH5N8_2.3.4.4 and rgH5N1_2.2.1.2. The three candidate vaccine strains were investigated in a comparative way for their immunogenicity, cross-reactivity, and effectiveness against currently circulating homologous and heterologous HPAI H5-type strains. The three vaccine formulae met all criteria specified by the World Organization for Animal Health (OIE) in terms of sterility and safety in two-week-old SPF chickens.

The generated vaccines showed an ability to induce protective HAI titers (≥2–5log2) against homologous HPAIV H5-type viruses but slightly lower HAI titers (≥2–3log2) by the second week post vaccination (WPV). These data are consistent with a recent study that used combined H5-type vaccines against H5N1 (clade 2.2.1.2) and H5N8 (2.3.4.4) in comparison with individual vaccine formulae [37]. The HAI titers increased gradually to be (≥6–9log2) and (≥2–8log2) against the heterologous HPAI H5-type strains at 4th WPV, indicating the immunogenicity and cross reactivity of raised antibodies. Unlike previous vaccination studies on avian influenza in poultry that showed high HAI titers [38], the immunization protocol of this study did not include any booster vaccination “second vaccine shot” to mimic the field application of the vaccine.

All vaccinated groups, challenged by homologous HPAI H5N1 viruses, were mostly or completely protected against clinical disease compared to challenged control groups, which died completely between days four and five post challenge infection. The average mortality of control chickens following challenge infection with HPAIV_H5N8_2.3.4.4 and HPAIV_H5N1_2.2.1.2 was shown to be four days post challenge infection [37]. The rgH5N1_2.3.4.4-vaccinated chickens had 100–80% protection against the two HPAIV H5Nx of clade 2.3.4.4 but 60% protection against the HPAIV_H5N1_2.2.1.2. However, the rgH5N8_2.3.4.4-vaccinated chickens had 100% protection against the two HPAIV H5Nx of clade 2.3.4.4 but 40% protection against the HPAIV_H5N1_2.2.1.2. On the other hand, the rgH5N1_2.2.1.2-vaccinated chickens showed 80% protection against homologous HPAIV_H5N1_2.2.1.2, but 60–40% protection against heterologous HPAIV H5Nx of clade 2.3.4.4. These findings indicate the optimum cross-reactivity of antibodies revoked against rgH5N1_2.3.4.4 to other tested homologous or heterologous clades. These data are consistent with the hypothesis that the rgH5N1_2.3.4.4 is sharing a similar hemagglutinin (HA) clade with the rgH5N8_2.3.4.4 and similar neuraminidase (NA) subtype/clade with rgH5N1_2.2.1.2 (Table 2).

Despite the fact that HA is the major influenza virus antigen, antibody (Ab) titers raised against N1 or N2 were shown to be accompanied by high NA enzymatic activity in vaccine preparations and improved virus neutralization capacity, suggesting that the NA content and activity of IAV vaccines predict immunogenicity [39,40].

Previous data were further confirmed by estimating viral shedding in vaccinated groups against control ones. The rgH5N1_2.3.4.4 was accompanied with a complete reduction in viral shedding at week seven post challenge with HPAIV_H5N1_2.3.4.4 and lower viral shedding against HPAIV_H5N8_2.3.4.4 and HPAIV_H5N1_2.2.1.2. This emphasizes the cross-reactivity of polyclonal antibodies raised against rgH5N1_2.3.4.4 and potentiate its possible application against the three tested challenging viruses.

## 5. Conclusions

In conclusion, the applicability of the generated vaccines against homologous circulating strains is significant in terms of complete clinical protection and significant reduction of viral shedding. What’s more, the cross-reactivity that was observed with the candidate vaccine strain expressing the surface glycoproteins from the newly emerging H5N1_2.3.4.4b further supports the value of its application as a multivalent vaccine intervention against the matching viruses/clades that were tested in this study.

## Figures and Tables

**Table 1:** Protection percent of the generated vaccines against different challenging HPAI H5-type viruses.

Challenge-Infection Strain	Group/Vaccine	Days Post-Infection									Number of Deaths	PROTECTION (%)
		1	2	3	4	5	6	7	8	9		
Challenge with HPAIV_H5N1_2.3.4.4	G1/rgH5N1_2.3.4.4										0	100%
	G2/rgH5N8_2.3.4.4										0	100%
	G3/rgH5N1_2.2.1.2				1			1			2	60%
	Control				2	3					5	0%
Challenge with HPAIV_H5N8_2.3.4.4	G1/rgH5N1_2.3.4.4						1				1	80%
	G2/rgH5N8_2.3.4.4										0	100%
	G3/rgH5N1_2.2.1.2				1		1	1			3	40%
	Control				2	3					5	0%
Challenge with HPAIV_H5N1_2.2.1.2	G1/rgH5N1_2.3.4.4						1	1			2	60%
	G2/rgH5N8_2.3.4.4							3			3	40%
	G3/rgH5N1_2.2.1.2								1		1	80%
	Control				2	3					5	0%

**Table 2:** Comparison of variable amino acid sequences related to vaccine protection in the HA1 of the three tested vaccine strains.

Amino Acid Residue *	H5N1_2.3.4.4b	H5N8_2.3.4.4b	H5N1_2.2.1.2
69	E	E	E
71	I	I	L
83	A	A	I
95	L	L	F
133	A	A	A
140	A	A	R
162	I	I	K
183	A	A	A
189	N	N	K
194	T	T	N
270	E	E	E

* H5 numbering excluding the signal peptide.

## Data Availability

The data presented in this study are available in the article.
